# The complete mitochondrial genome of yellow-bibbed lory, *Lorius chlorocercus* (Psittaciformes Psittacidae), with its phylogenetic relationship

**DOI:** 10.1080/23802359.2019.1687037

**Published:** 2019-11-11

**Authors:** Yun-Xia Chen

**Affiliations:** a Nanjing Forest Police College, Nanjing, P. R. China;; b Key Laboratory of Wildlife Evidence Technology State Forest and Grassland Administration, Nanjing, P. R. China

**Keywords:** *Lorius chlorocercus*, yellow-bibbed Lory, mitochondrial genome

## Abstract

In this study, the complete 17,855 bp mitochondrial genome of *Lorius chlorocercus* was obtained using sanger sequencing. It consists of 13 protein-coding genes, 22 tRNAs, 2 ribosomal RNAs, and a control region. The overall base composition is 22.5% T, 33.18% C, 30.42% A, and13.9% G. The phylogenetic tree was constructed using neighbour-joining (NJ) method based on 19 parrot species. Phylogenetic analysis showed that *L. chlorocercus* was closest to *Melopsittacus undulates*. The complete mitogenome data would be useful for further study on the molecular evolution of *L. chlorocercus*.

Parrots are charismatic birds, their plumage and capacity for learning make them highly sought after pets. The illegal trade in parrots poses a serious threat to the viability of native populations (Barber-Meyer 2010; Churgin et al. 2019). *Lorius chlorocercus* (yellow-bibbed lory), is mainly distributed in the eastern of the Solomon Islands (IUCN 2016). This species inhabits forests, tall secondary forests, densely planted vegetation areas, coconut gardens, etc. (IUCN 2016; Churgin et al. 2019). There is less information on the complete mitogenomes of the genus *Lorius*. Here, we characterized the complete mitochondrial genome of *L. chlorocercus*.

Whole blood samples of *L. chlorocercus* was collected from the individual bred in Nanjing Hongshan Forest Zoo (N32°09′, E118°80′), Jiangsu province, China. Genome DNA was extracted using the DNAiso reagent (Takara, Beijing, China) and stored in the Forest Police Forensic Center of State Forestry Administration (Accession S2019J1101204). PCR reaction and product purification were carried out by the Ex Taq and Minibest agarose gel DNA extraction kit (Takara, Beijing, China), respectively. Genome information was obtained through Sanger sequencing.

The complete mitochondrial genome (GenBank accession: MN515396) of *L. chlorocercus* is17,855 bp in length. The overall base composition of the genome is 22.5% T, 33.18% C, 30.42% A, 13.9% G, exhibiting an A + T bias content of 52.92%. The mitogenome consists of 22 transfer RNA genes, 13 protein-coding genes, 2 ribosomal RNA genes, and 1 control region. The structure and gene arrangement of the mitochondrial genome of *L. chlorocercus* is identical to other parrots (Eberhard and Wright 2016; Liu et al. 2019).

To assess the phylogenetic positions of *L. chlorocercus*, the phylogenetic tree was performed based on the complete mitogenome of 19 parrot species. Sequence dataset was aligned using ClustalX and analyzed using the neighbour-joining (NJ) method and the kimura 2-parameter model in MEGA version 7.0 (Temple University and Arizona State University), with 1000 bootstrap replicates (Kumar et al. 2016). Phylogenetic NJ tree showed that the mitogenome of *L. chlorocercus* was closest to *Melopsittacus undulates* (EF450826.1) (Figure 1). The genome information obtained here could contribute to further studies on the genetic diversity conservation of *L. chlorocercus*.

**Figure 1.**
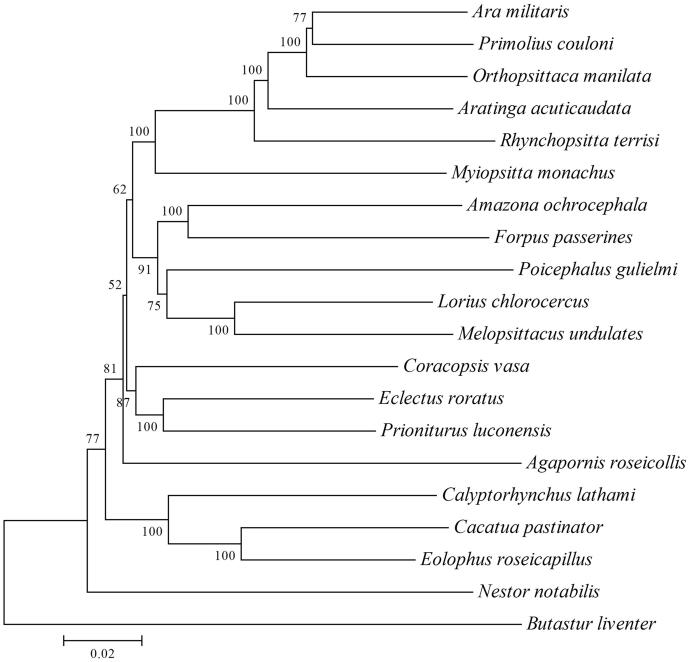
Neighbour-joining phylogenetic tree based on the complete mitogenomes of 19 parrots, constructed using MEGA 7.0. Ninety parrots species mitochondrial genomes have been deposited in the GenBank, the accession numbers are as follows: *Agapornis roseicollis* EU410486.1, *Amazona ochrocephala* KM611467.1, *Ara militaris* KM611466.1, *Aratinga acuticaudata* JQ782214.1, *Butastur liventer* AB830617.1, *Cacatua pastinator* NC_040142.1, *Calyptorhynchus lathami* JF414241.1, *Coracopsis vasa* KM611468.1, *Eclectus roratus* KM611469.1, *Eolophus roseicapillus* NC_040154.1, *Forpus passerines* KM611470.1, *Melopsittacus undulates* EF450826.1, *Myiopsitta monachus* NC_027844.1, *Nestor notabilis* MH133967.1, *Orthopsittaca manilata* KJ579139.1, *Poicephalus gulielmi* MF977813.1, *Primolius couloni* KF836419.1, *Prioniturus luconensis* KM611473.1, and *Rhynchopsitta terrisi* KF010318.1.
